# COVID-Associated Pulmonary Aspergillosis and Its Related Outcomes: A Single-Center Prospective Observational Study

**DOI:** 10.7759/cureus.16982

**Published:** 2021-08-07

**Authors:** Ahtesham Iqbal, Moazma Ramzan, Aftab Akhtar, Anam Ahtesham, Seemal Aslam, Javeria Khalid

**Affiliations:** 1 Critical Care, Shifa International Hospital, Islamabad, PAK; 2 Pulmonary and Critical Care, Shifa International Hospital, Islamabad, PAK; 3 Pharmacy, Bahawal Victoria Hospital, Bahawalpur, PAK; 4 Internal Medicine, Quaid-e-Azam Medical College, Bahawalpur, PAK; 5 Pharmacy, Shifa International Hospital, Islamabad, PAK

**Keywords:** covid associated pulmonary aspergillosis, invasive mechanical ventilation, severe covid pneumonia, outcome, probable capa

## Abstract

Background and objective

Invasive pulmonary aspergillosis (IPA) is a frequent complication among neutropenic patients. It is increasingly being reported in critical coronavirus disease 2019 (COVID-19) patients requiring ICU admission and invasive mechanical ventilation (IMV) and is known as COVID-associated pulmonary aspergillosis (CAPA). We conducted this large prospective observational study to determine the frequency of CAPA and its outcomes in the ICU population.

Methodology

This was a prospective observational study. We recruited 307 reverse transcription-polymerase chain reaction (RT-PCR)-confirmed cases of severe COVID-19 pneumonia requiring IMV. We excluded those who did not require IMV or had been transferred out to other hospitals. The Chi-square test was applied to find the association between categorical variables. A p-value of <0.05 was considered statistically significant.

Results

Out of the 307 cases of mechanically ventilated COVID-19 pneumonia, 61 had probable CAPA. The median age was 60 years. Malignancy and cirrhosis were significant risk factors associated with CAPA (p=<0.001, 0.001, respectively). *Aspergillus fumigatus* was detected in 78.7% of the cases. The median length of ICU stay was 11 days [interquartile range (IQR): 4-14]. Among CAPA cases, 70.5% developed septic shock and required ionotropic support. Among 61 probable cases of CAPA, 91.8% did not survive and there was a strong correlation between CAPA and ICU mortality (p=0.001).

Conclusion

We concluded that CAPA is a fatal complication of severe COVID-19 pneumonia and is associated with increased mortality.

## Introduction

Invasive pulmonary aspergillosis (IPA) is a fatal disease affecting immunosuppressed individuals with hematological malignancies and recipients of allogeneic bone marrow transplantation who develop acute graft versus host disease [1]. With the development of new treatment modalities and the emergence of novel diseases like influenza and coronavirus disease 2019 (COVID-19), the spectrum of risk factors of IPA has enormously expanded. In a study by Schauwvlieghe et al., influenza was found to be an independent risk factor for IPA, especially in patients who require ICU care [2]. Similarly, COVID-19 is also an independent risk factor for IPA and this entity is known as COVID-associated pulmonary aspergillosis (CAPA) [3]. Many risk factors have been identified to be involved in the pathogenesis of CAPA, which include the use of systemic corticosteroids in acute respiratory distress syndrome (ARDS), severe lung damage, comorbidities, and extensive use of broad-spectrum antibiotics [4]. Like influenza, COVID-19 also disrupts the respiratory epithelium and impairs ciliary dysfunction and the immune regulatory system, thereby facilitating the growth of *Aspergillus* spp. into tissues [5]. However, the pathogenesis of CAPA is still unknown. It is postulated that intense inflammatory response in COVID-19 results in a decline in lymphocyte count and impairs their function, which leads to increased susceptibility to CAPA in severe COVID-19 pneumonia [4]. The mortality rate associated with CAPA ranges from 66.1% to 80.6% [6]. The largest single-center prospective observational study conducted in the ICU population has reported 100% mortality in CAPA as compared to those without CAPA (44%) [7].

With the proven role of steroids in the treatment of severe COVID-19 pneumonia, fungal and bacterial coinfections are increasingly being reported in the ICU population and causing a huge impact on the outcomes of severe COVID-19 pneumonia. A meta-analysis of 24 studies comprising more than 3,000 severe COVID-19 pneumonia cases has reported 14.3% bacterial coinfections in ICU [8]. Similarly, CAPA is also increasingly being reported in the ICU population. Recently, 33% and 26% cases have been reported in France and Germany respectively [3,9,10]. A systematic review has reported a 35% to 3.8% incidence of CAPA in severe COVID-19 pneumonia, which included subjects mostly from European countries [11]. A Greek tertiary care referral hospital has reported an incidence of 3.3% in 179 polymerase chain reaction (PCR)-confirmed cases of COVID-19 pneumonia and, interestingly, none of them had immunosuppression [12]. Another study has reported a 27.7% incidence of probable cases after four days of invasive mechanical ventilation (IMV) [13].

Epidemiological studies conducted to evaluate the incidence of CAPA may not represent the true incidence of infection as most of them are small-sized and results are inconsistent in different parts of the world. Even though a retrospective study conducted at the national level reported 21.7% CAPA cases in 147 cases of COVID-19 pneumonia [14], the true incidence and mortality rates of CAPA remain underestimated in the ICU population since its results cannot be generalized to the larger population due to its small sample size. An expert panel has recently suggested conducting more epidemiological studies to evaluate the incidence of CAPA in the ICU population. In light of this, we conducted this prospective observational study to evaluate the frequency of CAPA and its associated outcomes in the ICU.

## Materials and methods

This was a prospective observational study conducted at the Medical ICU of the Shifa International Hospital, Islamabad, which is a large tertiary care center in Pakistan. The study period spanned from June 2020 to May 2021. Ethical approval was obtained from the Institutional Review and Ethical Committee of the Shifa International Hospital, Islamabad (IRB #: 158-978-2020). Written consent was taken from surrogates of study participants. After fulfilling inclusion and exclusion criteria, 307 cases were enrolled. The inclusion criteria were as follows: adult patients of ≥18 years, with RT-PCR-confirmed cases of COVID-19 pneumonia, admitted to the ICU with hypoxemic respiratory failure requiring IMV. We excluded those patients who did not require IMV, those who were RT-PCR-negative for COVID-19, those who did not consent to participate, and those who had transferred out to other hospitals. We followed up on our patients till the time of discharge from ICU or death.

Based on worsening respiratory symptoms with or without fever, we obtained chest X-rays and galactomannan for all patients along with other necessary investigations. Serum galactomannan testing was performed using Platelia™ Aspergillus kit (Bio-Rad Laboratories, Hercules, CA) with a cut-off value of >0.5. The tracheal aspirate was cultured for fungus. Probable CAPA was diagnosed based on worsening respiratory symptoms, radiological infiltrates, serum galactomannan >0.5, and fungal growth on tracheal aspirate. This definition was consistent with consensus criteria for research and clinical guidance for defining CAPA and proposed by the European Confederation of Medical Mycology/International Society for Human and Animal Mycology (ECMM/ISHAM) [15]. In addition to steroids, all patients received the usual standard of care including antibiotics. The infectious disease team was taken on board to decide about the inclusion of remdesivir, tocilizumab, or both. The decision for IMV was based on clinical condition and resuscitation code status. The mortality predictor scores like Acute Physiology and Chronic Health Evaluation (APACHE II), Sequential Organ Failure Assessment (SOFA), and Nutrition Risk in Critically ill (NUTRIC) scores were calculated at the time of admission to the ICU. All patients diagnosed with CAPA were treated with either oral voriconazole or amphotericin. Data including demographics and clinical, radiologic, and treatment outcomes were collected on a preformed structured questionnaire.

The primary measure was to evaluate the frequency of CAPA. Our secondary outcome was to evaluate mortality in CAPA. Mortality was defined as death during the stay in the ICU. Survival was defined as discharge from the ICU.

Statistical analysis

Results were analyzed using SPSS Statistics version 20 (IBM, Armonk, NY). The Shapiro-Wilk test was used to check the normality of continuous variables. Age, having normal distribution, was presented as mean ± standard deviation and other variables were presented as median [interquartile range (IQR)], being non-normal in distribution. Categorical variables like gender, comorbidities, diabetes mellitus, and outcomes were presented in percentages. A Chi-square test was applied to find the association between CAPA and ICU mortality. A p-value of <0.05 was considered statistically significant.

## Results

**Figure 1:**
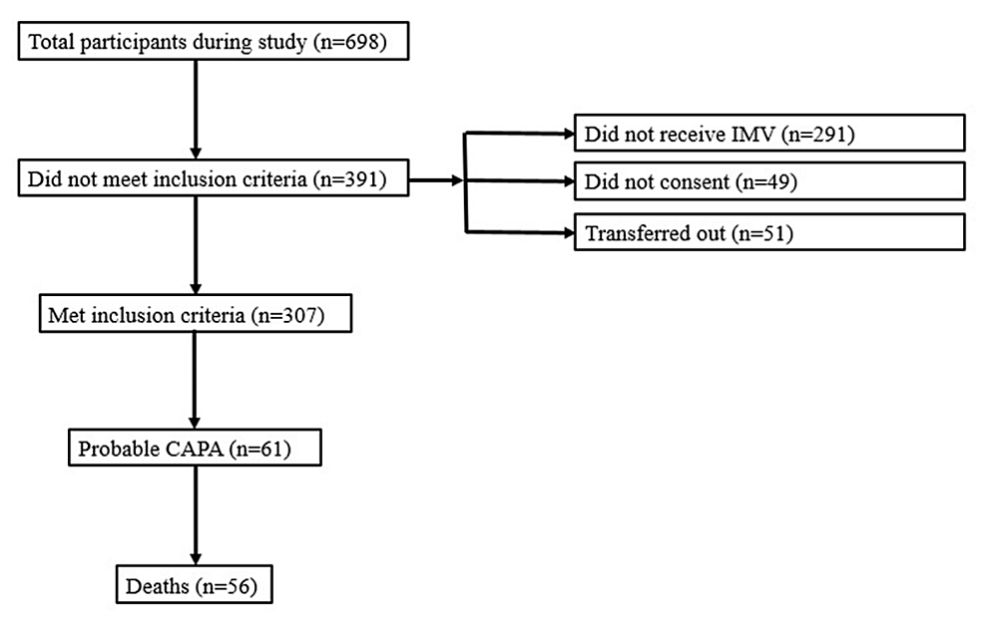
Flow diagram about the study participants CAPA: COVID-19-associated pulmonary aspergillosis; IMV: invasive mechanical ventilation

We prospectively analyzed 307 patients in total. The flow diagram about the study participants is presented in Figure 1.

The median age of our study participants was 60 years. Out of 307 participants, 19.9% (n=61) were diagnosed as probable cases of CAPA. Characteristics of probable CAPA cases are shown in Table 1. Out of these 61, 47.5% were females and 52.5% were males. Malignancy and cirrhosis of the liver were identified as statistically significant risk factors associated with CAPA (p=<0.001, 0.001, respectively), whereas diabetes mellitus and multiple comorbidities did not have a statistically significant association (p=0.55, 0.54, respectively). All probable cases of CAPA had received dexamethasone 6 mg/kg/day or methylprednisolone 1-2 mg/kg/day and also received voriconazole as a curative treatment. Mean values of clinical severity scores and characteristics of CAPA patients are given in Table 1. The culture of tracheal aspirates showed that *Aspergillus fumigatus* was the most common species to cause CAPA followed by *Aspergillus flavus* (78.7% vs. 16.4%); 57.4% of total probable CAPA cases received remdesivir and 54.1% were given tocilizumab. The median length of ICU stay was recorded as 11 days (IQR: 4-14). Among CAPA cases, 70.5% developed septic shock and required ionotropic support. We observed 60.9% (n=187) mortality among 307 study participants. Our results showed that 91.8% (n=56) did not survive in the cohort of probable CAPA, and there was a strong association between CAPA and mortality (p=0.001).

**Table 1:** Characteristics of probable CAPA cases CAPA: COVID-19-associated pulmonary aspergillosis; DM: diabetes mellitus; APACHE: Acute Physiology and Chronic Health Evaluation; NUTRIC: Nutrition Risk in Critically Ill; SOFA: Sequential Organ Failure Assessment; SAPS: Simplified Acute Physiology Score; ICU: intensive care unit; SD: standard deviation

Variables	Values (n=61)
Demographic data	
Age in years (mean ±SD)	60.7 ±8.7
Gender ratio (male:female)	29:32
Smoking history (n=80), % (n)	27.9% (n=17)
Hypertension, % (n)	49.2% (n=30)
Risk factors for CAPA, % (n)	
DM	73.8% (n=45)
Cirrhosis	8.2% (n=5)
≥2 comorbidities	90.2% (n=55)
Malignancy	21.3% (n=13)
Clinical variables	
APACHE II, median (IQR)	14 (10-21)
NUTRIC score, median (IQR)	4 (2-6)
SOFA score, median (IQR)	4 (4-7)
SAPS, median (IQR)	33 (27-45)
Aspergillus fumigatus, % (n)	78.7% (n=48)
Aspergillus flavus, % (n)	16.4% (n=10)
Aspergillus terreus, % (n)	3.3% (n=2)
Aspergillus niger, % (n)	1.6% (n=1)
Treatment and clinical course	
Remdesivir, % (n)	57.4% (n=35)
Tocilizumab, % (n)	54.1% (n=33)
Dexamethasone/methylprednisolone, % (n)	100% (n=61)
Voriconazole, % (n)	100% (n=61)
Lipid formulation amphotericin, % (n)	3.3% (n=2)
Vasopressors, % (n)	70.5% (n=43)
ICU days, median (IQR)	11 (4-14)
Death, % (n)	91.8% (n=56)

## Discussion

This was a prospective observational study that aimed to estimate the frequency of CAPA and associated mortality in our ICU population. Mean values of APACHE II, SOFA, NUTRIC, and SAPS II indicated the severity of disease in our study participants. We detected 19.9% cases of probable CAPA. The incidence of CAPA in our population was greater than the 10.1% rate reported in a retrospective study conducted in the USA. Many other studies from Europe have reported an incidence of 26.0% to 33% [3,9,10,13]. All these studies had a small sample size, and hence their results cannot be generalized to a larger population. Such variations in the incidence of CAPA are bound to happen due to variable infection control practices, ICU designs, and risk factors in the study populations. *Aspergillus* spp. is a common colonizer of the airway and can progress to overt infection in the ICU population because the use of antibiotics and steroid exposure can alter the host immune response [16-18]. IPA, an entity representing *Aspergillus* infection in non-COVID-19 patients, occurs most commonly in those who undergo chemotherapy for hematological malignancies. Our analysis showed that malignancy and cirrhosis were significant risk factors associated with CAPA infection. The association of malignancy and cirrhosis with the development of CAPA has not been adequately studied; CAPA is a relatively new entity and a lot of research is ongoing to explore its associated risk factors. A systematic review of 22 studies comprising only 85 patients has described the risk factors of CAPA and identified only two patients with malignancy [11]. It explains the scarcity of data and supports the work of Koehler et al., who has explored COVID-19 as an independent risk factor of CAPA [3]. Another important risk factor for the development of CAPA is tocilizumab, which is an interleukin 6 (IL-6) inhibitor and is known to cause multiple co-bacterial and co-fungal infections in the ICU population [19]. In our cohort of CAPA cases, 54.1% had received tocilizumab, and all had received corticosteroids, which are known to cause immunosuppression and paves the way for the growth of *Aspergillus* and tissue invasion to cause CAPA [20-23].

We observed 91.8% mortality in probable CAPA cases. It was beyond our study objective to explore factors associated with increased mortality in our cohort of probable CAPA cases. A meta-analysis of 28 studies has shown mortality of only 54.9% in the ICU population. This meta-analysis included 18 retrospective studies, and case definitions of CAPA were based on European Organization for Research and Treatment of Cancer/Mycosis Study Group (EORTC/MSG) criteria, which could have underestimated the incidence of CAPA as most ICU patients with CAPA lack traditional risk factors to meet the criteria of CAPA. In contrast, we diagnosed all cases of CAPA by satisfying the recent criteria proposed by Koehler et al. Another study from Italy has compared influenza-associated pulmonary aspergillosis with CAPA and has reported a mortality rate of 40-60% [24]. It is a subject of debate whether this mortality is attributed to CAPA itself or complicated COVID-19 pneumonia. A literature review was also inconclusive in this regard. A prospective study of 108 cases, after adjusting for confounding factors, has reported an increased association of mortality with CAPA (OR: 3.53) and a decrease in mortality with the use of antifungals [13]. In contrast, a large prospective US study did not find any association between increased mortality and CAPA despite poor outcomes in terms of length of stay and duration of IMV. Similarly, another case series of putative CAPA has shown decreased mortality even with the absence of antifungals. More robust evidence is needed to ascertain the impact of CAPA on the outcomes in the ICU population.

This study has some limitations. Firstly, it was a single-center study. Secondly, we did not study the contribution of CAPA to mortality in severe COVID-19 pneumonia as it was beyond our objective of the study. Finally, the galactomannan test in the bronchoalveolar fluid was not available at our study center, and hence we measured galactomannan in serum. Studies have suggested that the sensitivity of galactomannan is 40% as compared to that of the bronchoalveolar fluid, which is 90% [25]. This could have led to a potential bias of underreporting relating to the actual number of probable cases of CAPA in our study population. Larger comparative studies are required to calculate the impact of CAPA on mortality in ventilated patients. However, the large sample size of our study and its prospective design are the two major strengths of our study. Given our large sample size, our results can be generalized to critical COVID-19 patients requiring IMV.

## Conclusions

Based on our findings, CAPA is a frequent complication of severe COVID-19 pneumonia in the ICU population and is associated with an increased mortality rate. Prompt recognition and treatment may lead to favorable outcomes. We recommend further research to investigate its impact on ICU mortality in critical COVID-19 patients.
